# Personalized interpretable prediction of perceived sleep quality: Models with meaningful cardiovascular and behavioral features

**DOI:** 10.1371/journal.pone.0305258

**Published:** 2024-07-08

**Authors:** Max Moebus, Christian Holz

**Affiliations:** Department of Computer Science, ETH Zurich, Zürich, Switzerland; Portugal Football School, Portuguese Football Federation, PORTUGAL

## Abstract

Understanding a person’s perceived quality of sleep is an important problem, but hard due to its poor definition and high intra- as well as inter-individual variation. In the short term, sleep quality has an established impact on cognitive function during the following day as well as on fatigue. In the long term, good quality sleep is essential for mental and physical health and contributes to quality of life. Despite the need to better understand sleep quality as an early indicator for sleep disorders, perceived sleep quality has been rarely modeled for multiple consecutive days using biosignals. In this paper, we present novel insights on the association of cardiac activity and perceived sleep quality using an interpretable modeling approach utilizing the publicly available intensive-longitudinal study M2Sleep. Our method takes as input signals from commodity wearable devices, including motion and blood volume pulses. Despite processing only simple and clearly interpretable features, we achieve an accuracy of up to 70% with an AUC of 0.76 and reduce the error by up to 36% compared to related work. We further argue that collected biosignals and sleep quality labels should be normalized per-participant to enable a medically insightful analysis. Coupled with explainable models, this allows for the interpretations of effects on perceived sleep quality. Analysis revealed that besides higher skin temperature and sufficient sleep duration, especially higher average heart rate while awake and lower minimal activity of the parasympathetic and sympathetic nervous system while asleep increased the chances of higher sleep quality.

## 1. Introduction

Sleep quality drives the quality of life and affects productivity, mood and physical strength on the following day [1–3]. For individuals suffering from chronic diseases such as multiple sclerosis or Parkinson’s disease, sleep quality plays an even greater role as it can affect recovery after relapses [4, 5], pain management [6] and day-time fatigue [7]. Even in healthy people, sleep quality is an indicator for many sleep disorders and medical conditions [8–10]. Undetected and untreated sleep disorders significantly increase the chances of developing medical and psychiatric illness [11–14]. With potentially more than 40% of the population affected by sleep disorders [14], understanding the drivers of sleep quality and detecting sleep disorders early has become an ever more pressing issue.

To quantify characteristics of a person’s sleep, polysomnography (PSG) is the gold standard for measurements in controlled sleep studies, commonly conducted in specialized sleep laboratories [15]. During a PSG assessment, participants stay overnight, and a large array of high-quality signals is recorded [16]. Evaluated by trained medical personnel, PSG provides the most reliable data for research in the field of sleep science.

Due to the costs of PSG assessments, researchers have sought alternatives to laboratory environments to extend the quality assessment of sleep to a broader audience. Previous work has investigated the use of signals obtained from wearable devices to compensate for the expensive operation of sleep studies [16, 17], which are limited to few participants, often single-night recordings only [16, 18], and have unintended consequences for participants’ sleep behavior [15, 18, 19]. In contrast, running sleep studies during participants’ daily lives outside sleep laboratories without a study conductor being present at all times (in-the-wild) allows for cheaper and more representative assessments [20], allowing recordings from more participants [15] over longer periods of time [15, 20]. While signal quality and modalities are limited, the use of commodity wearable devices allows studies to be unobtrusive, allowing study participants to pursue their daily lives without much interference and follow their typical sleep routines.

In this paper, we introduce a novel method for estimating a person’s perceived quality of sleep based on signals obtained from today’s commodity wrist wearable devices, including smartwatches and fitness trackers. We demonstrate that by incorporating cardiovascular activity into the analysis, our method outperforms current estimators of sleep quality on in-the-wild recordings. Importantly, we restrict the input of features into our method to interpretable variables as well as our models themselves to post-hoc explainable techniques. By performing per-participant normalization, we maximize the interpretability of results and conclude with a medically insightful analysis.

### 1.1 Estimating perceived sleep quality based on smartwatch signals

Much previous work has focused on classifying sleep-wake states [15, 21–23] as well as sleep-stage classification [18, 24] based on wearable sensors. Using a wrist-worn accelerometer alone, actigraphy devices have emerged as reliable sleep-wake classifiers [25]. Sleep staging is the arguably more difficult problem and previous work has often utilized multiple sensors such as photoplethysmography in addition to an accelerometer [26, 27].

While clearly complementary, few prior efforts attempted to model perceived sleep quality using wearable sensors [9, 28–30]. Past longitudinal studies concerned with perceived sleep quality are mainly smartphone-based and have used information about smartphone usage [29, 30], social interaction and physical activity [28]. A study utilizing a wrist-worn wearable used skin temperature and electrodermal activity in addition to wrist movement to model perceived sleep quality [9]. However, prediction accuracies for personalized models were rather unsatisfactory with 57.3–61.5% in a binary setting [9]. Without any previous knowledge about participants, prediction accuracies were particularly low with a balanced accuracy of 46.8–52.7%.

Modeling sleep quality based on the characteristics of a person’s cardiovascular physiology has been curiously absent, despite the availability of corresponding sensors in most wrist-worn wearables. In addition to heart rate, heart rate variability especially has been found to vary greatly between different sleep stages [31–33]. Heart rate variability is an indicator of the autonomic nervous system (ANS), which is impacted by an individual’s response to stress [34] and whose activity considerably impacts next-day mental performance [35]. Further, heart rate and heart rate variability are also significantly affected by physical exercise, diet, and emotional states such as excitement or anxiety [36].

We introduce a novel method that estimates perceived sleep quality from aggregated sensor recordings collected while asleep and during the previous day. As input into our estimation model, we extract simple and *interpretable* features from the wearable sensor streams, including sleep duration, cardiovascular activity (heart rate and heart rate variability), actigraphy counts (derived from wrist movement), skin temperature and electrodermal activity. We utilize the M2Sleep dataset [9], which comprises continuous smartwatch recordings from 16 participants over 30 days. To the best of our understanding, the M2Sleep dataset is unique in combining perceived sleep quality responses and continuous blood volume pulse recordings in an intensive longitudinal study. Specifically, participants wore an E4 watch that recorded activity data from an accelerometer (IMU), body skin temperature (TEMP), blood volume pulses (BVP), and electrodermal activity (EDA). Our evaluation demonstrates that in combination with features about skin temperature and actigraphy, features from heart rate and heart rate variability boost model performance to up to 70% accuracy and reduce the error in direct comparison with related work by 36%. We conclude with a discussion of our results and their implications for medical use.

Taken together, we contribute a new approach to model perceived sleep quality using interpretable features that we normalize per-participant highlighting the importance of cardiac activity to model perceived sleep quality and outperforming previous work. Due to the simplicity of the used features, a transformation of the modeling problem, and the post-hoc interpretability of our used model, our approach allows us to interpret the results. We believe that our approach generalizes to other use-cases where subjective responses are modeled in in-the-wild studies involving wearable sensor data.

## 2. Related work

While sleep phases are clearly defined [37–39], sleep quality is defined rather poorly [40, 41] and subject to high inter- as well as intra-individual variation [42]. Objectively, sleep quality is often assessed using sensor recordings from inside sleep laboratories, for instance the proportion of time spent in different sleep stages [43]. Perceived (aka. subjective) sleep quality is assessed via questionnaires and the Pittsburgh sleep quality index [44] (PSQI) has emerged as a widely used questionnaire for perceived sleep quality [45]. Wearable devices, especially when coupled with frequent questionnaires, combine the advantages of objective and subjective assessments and allow to link objective sensor measurements to subjective assessments of sleep quality during longitudinal studies [38, 39].

### 2.1 Sleep laboratories

Over recent decades, sleep experiments and studies have been conducted mainly inside sleep laboratories [39]. The setting inside sleep laboratories referred to as polysomnography (PSG) has become the gold standard of sleep science [41]. PSG allows to utilize a broad array of sensors at high quality, including electroencephalograms (EEG), electrocardiograms (ECG), microphones, cameras, electromyography (EMG), breathing belts, sphygmomanometers, and more [41]. Due to the array of equipment involved, needed space and personnel, PSG-based studies are expensive [17].

### 2.2 Objective sleep quality

Objective sleep quality often refers to factors that enable a ‘healthy night’ of sleep [46]. This includes sleep duration, sleep continuity, time spent in different sleep stages, sleep efficiency and sleep latency [43, 47, 48]. Sleep disorders refer to the case when sleep is chronically worsened, such as insomnia [49], sleep apnea [50] or periodic limb movement disorder [51]. The diagnosis of sleep disorders such as sleep apnea, for instance, is conducted in sleep laboratories [17]. While PSG might be used for the final assessment of sleep disorders, questionnaires measuring subjective sleep quality are often sufficient for initial screening [52]. At-home assessments using validated (wearable) sensor setups have also become increasingly commonplace to detect sleep disorders, which further demonstrates the value of wearable sensors in decreasing patient burden and costs at the same time [53].

### 2.3 Questionnaires & perceived sleep quality

Sleep studies conducted using questionnaires assess perceived sleep quality and reconstruct information collected in sleep laboratories. Developed in 1989, the Pittsburgh sleep quality index [44] (PSQI) is one of the most widely used sleep quality questionnaires [45]. In addition to answering questions about perceived sleep quality on a Likert scale, users are also asked about objective sleep quality metrics such as sleep duration, sleep continuity, or sleep onset. While a subjective assessment of sleep duration was found to better predict perceived sleep quality than actual sleep duration [54], subjective assessments of sleep duration were found to differ on average by 29 minutes compared to actual sleep duration [55]. Variables collected using questionnaires, thus, must be treated carefully. This highlights the advantage of a combination of wearable sensors and questionnaires to model perceived sleep quality using objectively recorded data.

### 2.4 Wearable devices

#### 2.4.1 Sleep staging & sleep-wake classification

Sleep staging aims to approximate the time spent in different sleep stages. Using wearable devices, past literature has attempted to match the performance of PSG-based methods [21, 38]. Solely using the wrist’s movement, actigraphy constructs an activity count to distinguish between wakefulness and sleep [56] and has emerged as a commonly used tool in sleep science [22]. Some commercially available actigraphy devices include the Actiwatch2, Actiwatch Spectrum and Actiwatch Spectrum Plus by Phillips, as well as the wGT3X-BT by Actigraph, all of which were found to produce results correlating strongly with PSG-derived measurements [25].

Smartphone-based approaches for sleep-wake classification and sleep staging include work by Gu et al. [34], Saeb et al. [57] and Cuttone et al. [23]. While Cuttone et al. [23] base their sleep-wake predictions solely on smartphone usage, Saeb et al. [57] further include data about location, motion, light and sound. The sleep stage detection system Sleep Hunter [24] utilizes a smartphone lying next to users’ heads while asleep to extract body motion and acoustic events while asleep based on the smartphone’s microphone and accelerometer. Coupled with demographic information, Gu et al. [24] thus distinguish between light, deep and REM sleep.

More recently, wrist-worn devices that collect information about biosignals such as heart rate have been used more frequently for sleep-wake classification and sleep staging [21, 38]. On consumer-grade devices from Fitbit or Apple Inc., Walch et al. [26] and Beattie et al. [27] distinguish between different sleep phases using heart rate, heart rate variability and wrist movement. Gashi et al. [9] distinguish between periods of sleep and wakefulness using the clinical-grade Empatica E4. In contrast to the two previously mentioned methods, Gashi et al. [9] did not make use of heart rate and heart rate variability, but of wrist movement, electrodermal activity and skin temperature.

#### 2.4.2 Perceived sleep quality modeling

Perceived sleep quality has been modeled using a broad array of data including activity, social interactions, mood, smartphone usage, light exposure, skin temperature and electrodermal activity. We identified four papers that have attempted to model perceived sleep quality for consecutive days using variations of wrist-worn wearable sensor data or aggregates thereof [9, 54, 58, 59]. A further three papers attempted the same only using data collected from smartphones [28–30].

Niemeijer et al. [30] modeled the perceived sleep quality of 60 participants for two weeks using a smartphone recording acceleration, activity, charging status and WIFI status. Bai et al. [28] modeled the perceived sleep quality of 15 participants across 30 days using information about previous nights’ sleep quality as well as features related to social and physical activity throughout the day derived from a smartphone. Using data about participants’ smartphone app usage, the physical environment and social interactions, Jayarajah et al. [29] modeled the perceived sleep quality of 400 students for on average 66 days per participant.

For modeling perceived sleep quality, Goelema et al. [54] compared the information content of subjective assessments of sleep duration and sleep continuity to objective assessments based on actigraphy data. SleepGuard (developed by Chang et al. [58]) tracks sleep stages and models daily perceived sleep quality responses using a single smartwatch. The collected information includes body posture and movements, acoustic events, and illumination conditions. To analyze potentially bidirectional relationships between symptoms of multiple sclerosis patients, Guo et al. [59] analyze subjective sleep quality based on information about activities of daily living as well as objective sleep quality metrics derived through a wrist-worn fitness tracker. Daskalova et al. [60] assess how recommendations about sleep and exercise affect daily perceived sleep quality and objective sleep quality metrics derived using a fitness tracker. On the M2Sleep dataset, Gashi et al. [9] collected data about participants’ wrist movement, electrodermal activity and skin temperature to model sleep quality as a binary response. While all of the related work mentioned above includes a broad array of signals to model perceived sleep quality, an analysis based on cardiac activity is absent.

### 2.5 Explainability & interpretability

For applications in the medical domain, the trade-off between performance and interpretability as well as explainability must be evaluated carefully. So called ‘black-box’ models such as neural networks tend to outperform linear techniques such as logistic regression at binary classification [61]. However, to assess risks, establish confidence in predictions and validate methods, explainability and interpretability of computational methods play a great role in the medical domain [62]. Despite growing interest in deep learning techniques over recent years, so called ‘glass-box models’ such as linear regression or shallow decision trees have also experienced growing interest as part of the growing field around explainable AI (*X*AI) [63]. For models that are inherently explainable, such as tree ensemble methods [64], methods for post-hoc feature analysis have been proposed such as individual conditional expectation (ICE) and partial dependence plots [63] (PDPs).

## 3. Dataset, signal processing, and interpretable feature extraction

We now describe the dataset we incorporate in the design and evaluation of our estimation method for perceived sleep quality. We build on an existing dataset, process signals and extract novel features, and compare our method’s performance using the original features with the prediction performance using our novel features. We place particular importance on deriving simple and physiologically interpretable features, which allows us to better understand their importance as drivers of sleep quality. Thus, we aim to further enhance the understanding of sleep and sleep quality.

### 3.1 Source dataset

**Fig 1:**
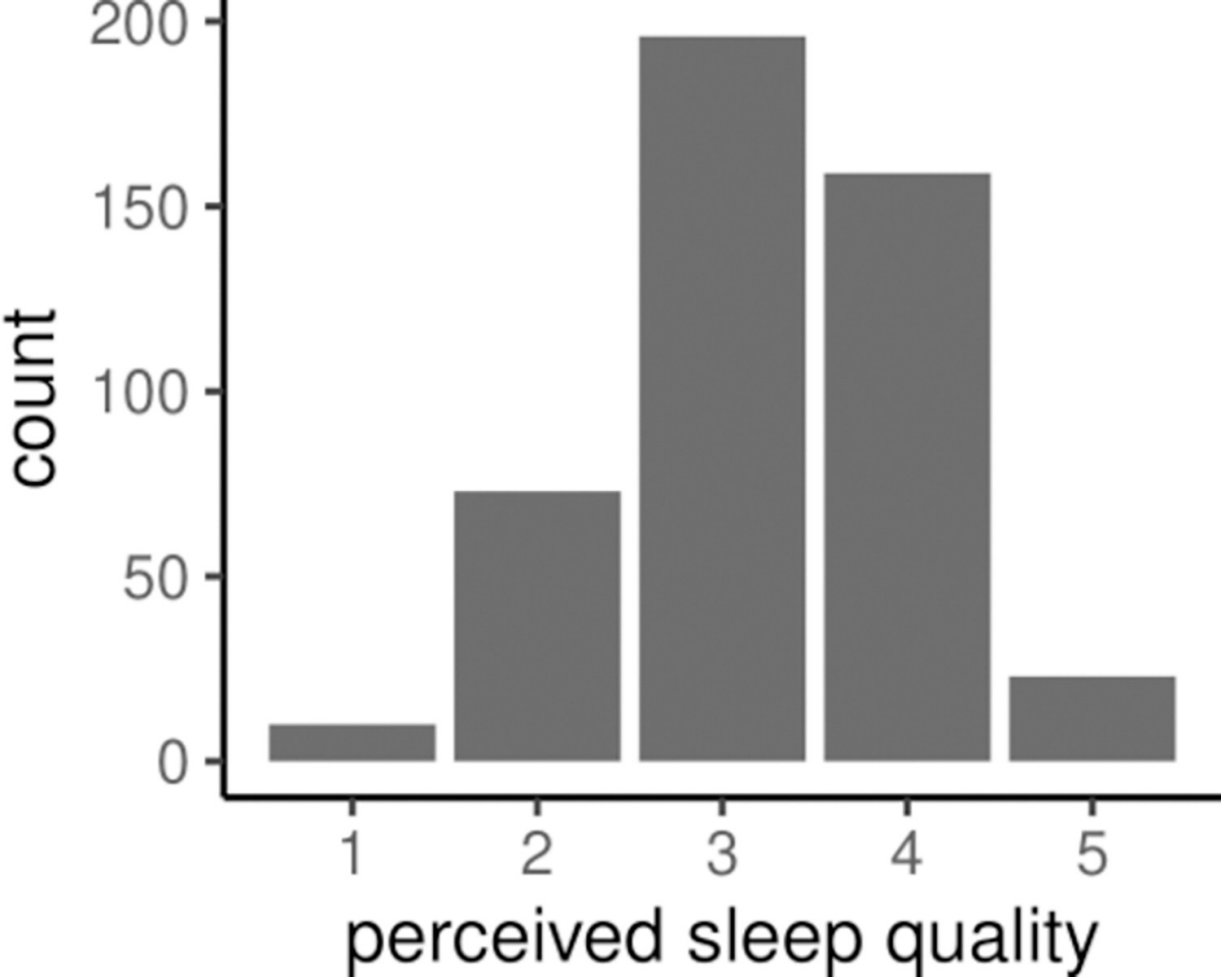
Distribution of the sleep quality response (Table 1).

We first process the signals recorded in the M2Sleep dataset [9]. All ethical and experimental procedures and protocols of this study were reviewed and approved by the ethics committee of the Faculty of Informatics at the Università della Svizzera italiana (ID: 2021-04-20-INF-A). All participants provided written informed consent. We gained access to the anonymized data on 13^th^ of April 2022. The corpus comprises recordings over 30 days from 16 participants (5 female, 11 male), ages 19–35 years (avg = 26.4, sd = 4.5). Study participants wore an Empatica E4 watch, a wearable collection apparatus for biosignals. The Empatica E4 was validated in various studies for its heart rate, heart rate variability and electrodermal activity measurements [65–67]. In addition, participants rated their perceived sleep quality each morning after waking up on a five-point Likert scale as shown in Table 1. Fig 1 shows the distribution of sleep quality across the 30 days. The average response was 3.25. Participants did not record whether they had any sleep disorders. Participants did, however, fill out the Pittsburgh Sleep Quality Index (PSQI) questionnaire [44]. Participant S13 recorded that they had trouble falling asleep within 30 minutes at least three times a week. As outlined in Section 3.3, participant S13 had to be excluded for the modeling process due to too many observations with missing data.

**Table 1:** Sleep quality Likert scale.

Score	Answer
5	Excellent
4	Good
3	Normal
2	Poor
1	Very poor

The 16 participants rated their perceived sleep quality on this five-point Likert scale each morning of the 30-day period.

No other participant indicated potential sleep disorders in their response to the PSQI. With 16 participants, the dataset is not representative of a broad population. While we use it to establish the importance of cardiac features for perceived sleep quality prediction and to showcase the predictive performance of an approach generating interpretable results, the results are unlikely to be robust towards individuals suffering from sleep disorders, or who differ too much to the study population.

Participants wore the E4 watch on the non-dominant wrist during the entire night. They put on the watch at least 4 hours before going to sleep and took it off not before at least 4 hours after waking up had passed. The watch continuously recorded participants’ blood volume pulse (BVP, 64 Hz), their electrodermal activity (EDA, 4 Hz), the 3-axis acceleration of the wrist (IMU, 32 Hz), as well as participants’ skin temperature (TEMP, 4 Hz).

### 3.2 Data processing and feature extraction

From each participant’s recordings, we extracted several features from the E4’s logs of BVP, EDA, IMU, and TEMP in addition to the duration of participants’ sleep and their sleep onset. We selected interpretable and simple features from the time domain in order to increase the interpretability of the results and enable an analysis regarding the drivers of sleep quality.

#### 3.2.1 Cardiac activity from the blood volume pulse (BVP)

Based on the BVP signal, features about the cardiovascular activity can be inferred such as heart rate (HR) and heart rate variability (HRV) [68]. HR and HRV vary between different sleep stages and are affected by factors such as stress, arousal, activity levels, or anxiety [69–72]. All of these were found to affect sleep quality [73–77]. HRV further measures the activity of the autonomic nervous system, which varies between different stages of sleep. The autonomic nervous system affects many of the body’s involuntary functions and its activity was found to be a predictor of general mortality [78]. Some parts of the autonomic nervous system are more active while asleep than at any time during wakefulness [70]. Sajjadieh et al. [35] further found evidence for a bi-directional relationship between sleep quality and HRV.

We processed BVP for HR and HRV computation from the E4’s recordings in two separate ways to compare prediction performances. In the E4 condition, we directly used the inter-beat intervals (IBIs) between heartbeats as calculated by the Empatica E4. The Empatica E4 automatically removes IBIs where it detects evidence of motion artifacts [79]. We therefore maintained all HR and HRV values from 5-minute windows without any missing IBIs. We did not interpolate IBIs for missing windows, because interpolation has been found to significantly affect HRV metrics [80].

In the processed condition, we derived cardiac features from the raw BVP signal recorded by the E4 to increase the number of 5-minute windows with information about HR and HRV. We first filtered the raw BVP signal using a Chebychev Type 2 4^th^ order bandpass filter with cutoff frequencies of 0.5 and 10 Hz as common for short BVP signals [81]. We then extracted features from every 5-minute window with 2.5-minute overlap. Using HeartPy [82], we derived the IBI values for each window. We discarded all windows where HeartPy reported low signal quality, retaining only 5-minute windows of valid HR and HRV values as in the previous condition.

In both conditions and for each retained 5-minute window of IBIs, we extracted participants’ heart rate (HR) as well as heart rate variability (HRV) expressed through the following features: root mean square of successive differences of heartbeat (RMSSD), the standard deviation of the inter-beat-intervals (SDNN), and the standard deviation of distance from −45° line of points-caré plot of consecutive IBI (SD2). We adjusted all HRV metrics to participants’ gender, age, and the respective time-of-day since these factors were found to significantly affect HRV making a comparison of different values difficult [83]. For each of these metrics, we computed 6 simple features: mean, maximum, and minimum, each while during the awake phase and during the asleep phase.

#### 3.2.2 Autonomic function from electrodermal activity (EDA)

EDA is a measure for activity of the sympathetic part of the autonomic nervous system, which varies during different sleep stages—similar to HRV [84]. Sano et al. [84] found an increased number of spikes of EDA and the highest overall amplitude during deep sleep. EDA might thus be an indicator for intensity of deep sleep potentially linked to sleep quality.

We used multiple conditions to validate the EDA signal as proposed by Nasseri et al. [85]. We removed any interval where the amplitude of the EDA signal was below 0.05 *μS*, since this usually implies that the sensor did not have skin contact [85]. Additionally, we removed any second where the amplitude of the EDA signal did not change by more than 0.01*μS*, since this implies that the EDA sensor did not measure at all [85]. The naturally possible range of change of skin conductivity lies within [−0.1Δ*tx*, 0.2Δ*tx*] across any interval of length Δ*t* starting at *t*_0_, if the signal had amplitude *x* at *t*_0_ [86, 87]. Hence, we also removed any interval where the amplitude increased by more than 20% or decreased by more than 10% per second. Based on the remaining data, we extracted the overall mean, maximum and minimum while awake and asleep as well as the average, maximal and minimal slope while awake and while asleep.

#### 3.2.3 Wrist motion from the accelerometer (IMU)

Movement while asleep can be an indicator of reduced sleep continuity or potentially also sleep disorders such as periodic limb movement disorder [51]. Sleep continuity, in particular, was found to predict also subjective sleep quality by Della Monica et al. [88]. Based on the signal of the accelerometer, we followed past literature to calculate activity counts [89]. Using Sadeh’s and the Cole-Kripke algorithm [22, 90], we estimated the participants’ sleep and wake times. We estimated the beginning of the sleep phase as the first minute of a 10-minute window where participants were estimated to be asleep after the time they reported they went to bed. We calculated the sleep onset as the difference between the participants reporting that they went to bed and their estimated falling-asleep time. Similarly, we calculated when participants woke up, looking in a one-hour window around the time they reported they woke up. Generally, we averaged the results we got when applying Sadeh’s and the Cole-Kripke algorithm. We aggregated the actigraphy counts while participants were awake and asleep as a measure of physical activity throughout the day and sleep continuity during the night. Both Sadeh’s and the Cole-Kripke algorithm have shown acceptable performance in past studies but are not exact [91, 92].

#### 3.2.4 Skin temperature (TEMP)

Skin temperature is affected by body temperature as well as room temperature [93]. It thus provides information about the circadian rhythm since body temperature has to drop to enable sleep, which causes skin temperature to rise to enable the cooling of the body’s core temperature [94]. As the interface between the body and the environment, the skin’s temperature also provides information about the room’s temperature that the participant is sleeping in, which was found to affect sleep quality and lead to disrupted sleep in the summer [95, 96].

We used features calculated on the raw temperature signal as input for our modeling process as proposed in previous literature [9]. We calculated the mean, maximum and minimum temperature while awake and while asleep as well as the slopes across 5-minute windows with 2.5-minute overlap, and computed the mean, maximum and minimum slope while awake and asleep. This resulted in a total of 12 features based on the recorded temperature signal.

### 3.3 Data set construction & removed observations

We constructed 2 datasets: dataset A and dataset B. The difference between the two datasets lies in the way we calculated features related to HR and HRV. All other features are identical. For dataset A, we used the IBIs supplied by the E4 to calculate HR and HRV (E4 condition). We removed any observations with missing values. This led to 241 out of 463 observations being removed due to IBIs not being recorded for any 5-minute interval while the participants were awake or asleep and, thus, no HR and HRV data being available for the awake phase. This fully removed participant S13 from the dataset. This left a total of 222 observations for 15 users, leading to an average of 14.8 data points per user.

**Fig 2:**
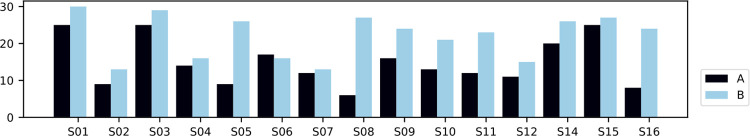
Number of observations per participant after removing missing data. In dataset A, we calculated heart rate variability and heart rate based on 5-minute windows of consecutive IBIs as supplied by the Empatica E4. For dataset B, we computed IBIs based on the filtered BVP signal using HeartPy [82].

We constructed dataset B by calculating HR and HRV features based on 5-minute intervals where we calculated the IBIs based on the filtered BVP signal using HeartPy (Processed condition). Again, we removed participant S13 from the dataset since no information about HR and HRV was available. After we removed any observation with missing values, dataset B was of size 330 and consisted of 15 users. The number of observations included in the two datasets per participant is displayed in Fig 2.

## 4. An interpretable method for estimating perceived sleep quality

Due to the imbalance of the 5 levels of responses (Fig 1), we modeled perceived sleep quality as a binary response. We grouped together responses 4 and 5, as well as 1–3. This resulted in a nearly balanced dataset with a split of 53% versus 47%. We refer to this as modeling absolute perceived sleep quality. Even though the mean response of 3.25 was higher than the ‘normal’ response, using ≥ 4 as a cut-off point corresponds to modeling whether an individual reported high sleep quality.

Due to the differences in the distribution of the response and input features per participant as we discuss in detail in Section 4.2.2, we normalized the perceived sleep quality responses as well as the features per participant in a second modeling step. We then transformed the now normalized response again into the binary setting, based on the participants’ average sleep quality. Thus, we modeled if a participant slept at least as well as their average. We will refer to this as modeling normalized perceived sleep quality.

### 4.1 Explainable machine learning methods

To enable an analysis of the drivers of perceived sleep quality while keeping predictive performances high, we required an at least post-hoc explainable machine learning technique to be used on interpretable features. We modeled perceived sleep quality on each of the 2 datasets using a tree ensemble method [97]. Tree-based methods inherit the interpretability and explainability of decision trees [64] and have demonstrated high predictive performances for medical applications [63, 98, 99]. However, they are less interpretable than so-called ‘glass-box models,’ such as linear modeling techniques or a single shallow decision tree, and require post-hoc visualization to interpret features and reconstruct the decision function [63, 98]. Thus, we conducted our analysis of the drivers of perceived sleep quality based on partial dependence plots (PDPs). The audience who can interpret the results of our approach is still restricted. Primarily, the approach is designed so that we ourselves, the authors of this paper, can interpret its results.

To adequately assess the performance of our classifier, we cross-validated the performance of all models using leave-one-out cross-validation (LOO-CV). At each iteration of the LOO-CV, we left one participant out of the training set and, thus, were able to evaluate the model on a previously unseen individual. We repeated each LOO-CV ten times. Thus, to evaluate any model each user was left out 10 times, totaling 150 cross-validation splits. Each test and train dataset was balanced by randomly removing from the over-represented class. We evaluated models using prediction accuracy, micro-F1 score and the area under the receiver-operating curve (AUC). For the full modeling and evaluation process, we used Python version 3.8.8 [82].

When modeling absolute perceived sleep quality, we evaluated the performance of our model in comparison to a baseline set by Gashi et al. [9] When modeling normalized perceived sleep quality (see Section 4.2.2 for further detail), with a focus on interpretability, we compare our model against ‘glass-box models’ that are fully interpretable and explainable, namely a generalized linear model (GLM) and a generalized additive model (GAM). For logistic regression, GLMs and GAMs both naturally model the odds ratio of observing higher than average sleep quality per participant. Due to their high interpretability, GLMs and GAMs are commonly used in the medical domain as well as the environmental sciences [100].

### 4.2 Models: As recorded and per-person normalization

**Fig 3:**
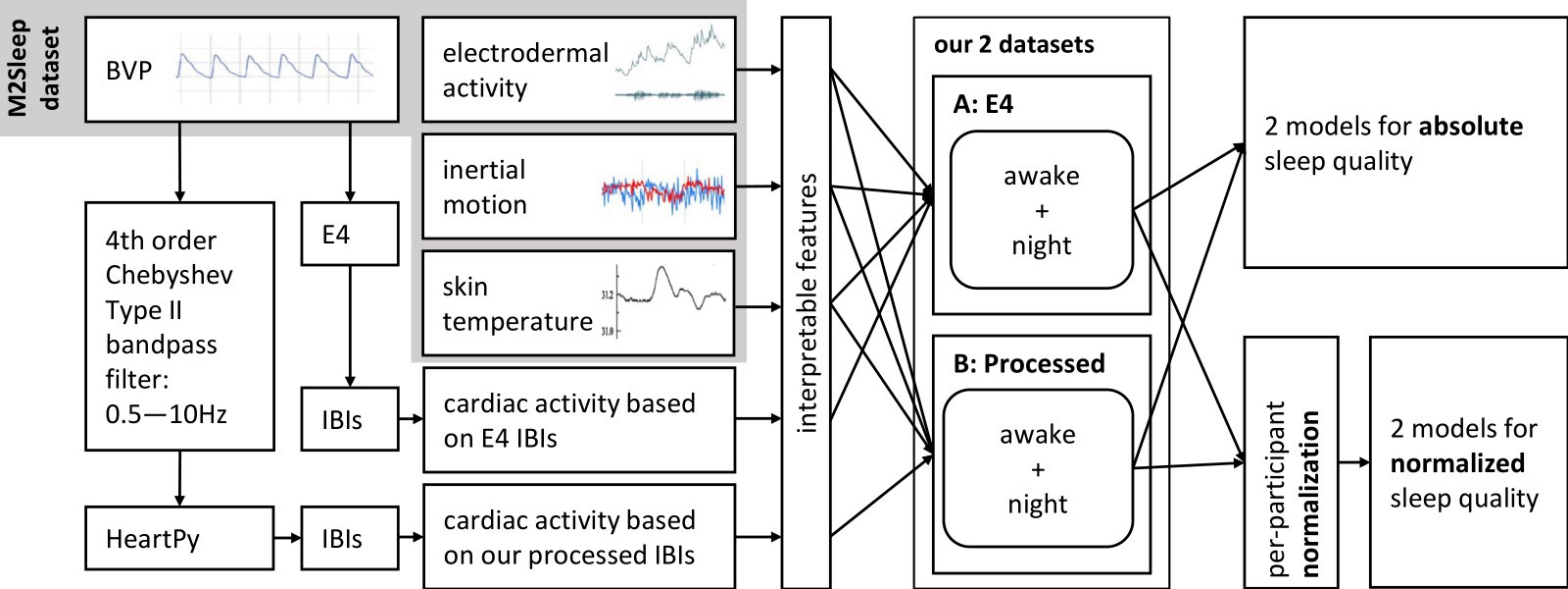
Visualization of the data processing and modeling process. We analyzed absolute perceived and normalized perceived sleep quality based on two datasets each. Dataset A and B differ in the way we calculated inter-beat-intervals based on the Blood Volume Pulse (BVP) signal. Both datasets consist of simple and interpretable features related to cardiac activity, skin temperature, electrodermal activity and physical activity.

In addition to modeling sleep quality responses as reported by participants each morning, we separately adjusted responses and signals in order to analyze how relative changes in signals per participant can predict relative changes in perceived sleep quality. Through this procedure, we obtained two versions of each of the two datasets: one version corresponding to the scenario where we modeled perceived sleep quality as recorded (referred to as *absolute perceived sleep quality* from now on) using the features as recorded and a second version where we modeled perceived sleep quality normalized per participant (*normalized perceived sleep quality*) using adjusted features as outlined below in Section 4.2.2. As we outline in more detail in Sections 4.2.3–4.3.4, this results in greater interpretability and increases the information content of predictions. A visualization of the constructed datasets is displayed in Fig 3.

#### 4.2.1 Reported sleep quality modeled through signals as recorded

For absolute perceived sleep quality, similar to the work of Gashi et al. [9], we modeled absolute perceived sleep quality as a binary response, using ≥ 4 as a cut-off point between high and low sleep quality (see Table 1). The respective results for this scenario are listed in Section 5.1

#### 4.2.2 Normalized sleep quality modeled using features normalized per participant

In the second scenario, we modeled normalized perceived sleep quality as a binary response using normalized input features. To normalize input features and the response, we subtracted the average value per participant and divided by the standard deviation. We transformed the response into the binary setting based on whether a participant slept at least as well as their personal average. The results of this scenario are described in Section 5.2. In Section 4.2.3–4.3.4, we outline why normalizing the response per participant provides more value for participants and why the per-participant normalization of features allows for better medical interpretation.

#### 4.2.3 Problematic per-user distributions of the response

**Fig 4:**
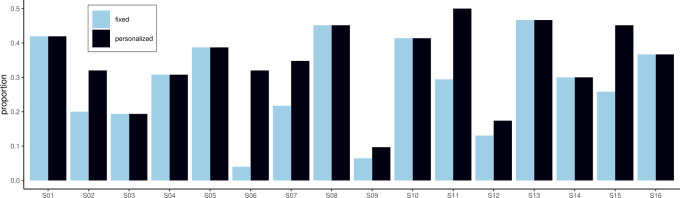
Proportions of the under-represented label per participant. Personalized cut-off points to distinguish between high and low perceived sleep quality reduce the imbalance of the two sleep quality labels compared to a population-based cut-off point.

While the dataset overall is nearly balanced between high and low sleep quality when using ≥ 4 as the sleep quality threshold (47% versus 53%), the two sleep quality labels are less balanced per individual participant. When splitting based on ≥ 4 for the whole population, the under-represented labels make up 4.0–46.7% of observations, depending on the participant. Imbalanced classes not only make training more difficult but also provide less value to users since in the case of participant S06, for instance, only 4.0% of predictions would differ from the mode of predictions (if the model achieved perfect accuracy). Fig 4 highlights the imbalance between the two different labels per participant.

Using the participants’ average responses as the sleep quality threshold to decide between low- and high-quality sleep, decreases the imbalance in 7 out of 16 cases, and results in the same level of imbalance otherwise. In the case of participant S09, the proportion of the under-represented label only increases to 9.3%, which is due to the mode response of participant S09 making up 86.7% of their responses.

#### 4.2.4 Problematic per-user distributions of the input features

In addition to a problematic distribution of the absolute perceived sleep quality response per participant, input features that relate to biosignals such as heart rate also require per-participant normalization to become more interpretable. Resting heart rate, for instance, was found to differ by up to 70 BPM between individuals and differs due to age as well as gender [101]. However, per individual, the resting heart rate showed to be very consistent and only differed slightly on a day-to-day basis [101].

**Fig 5:**
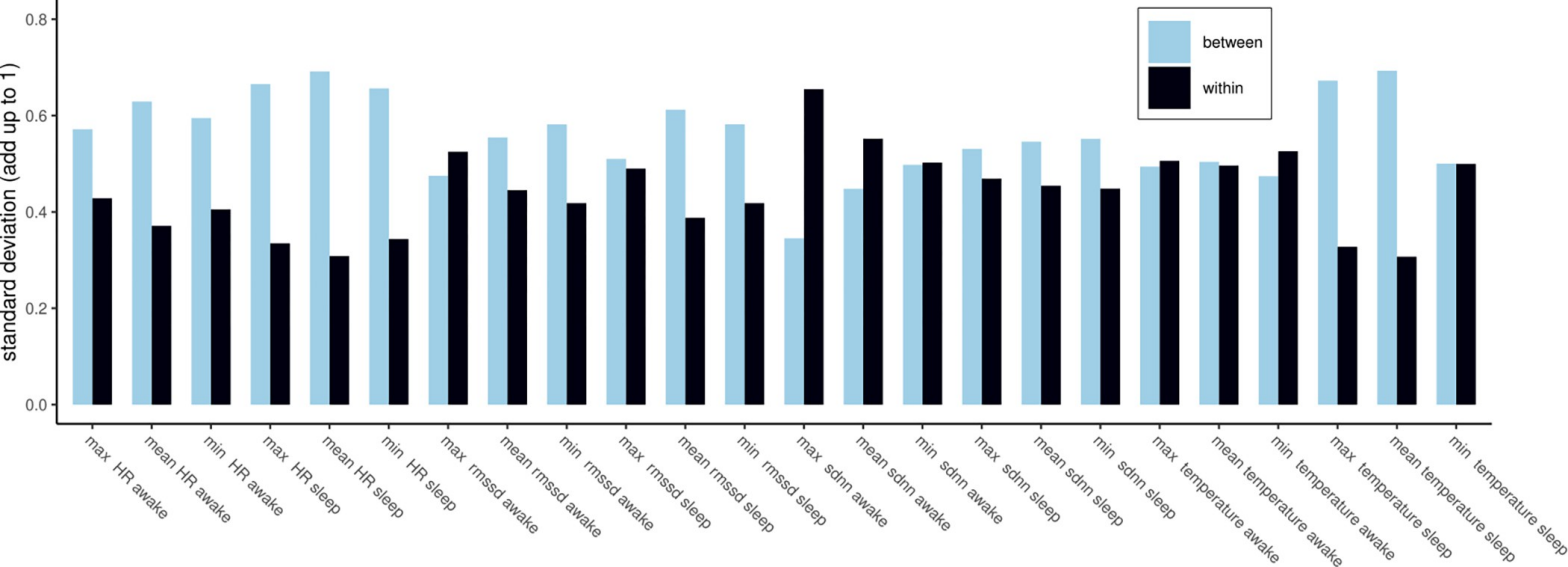
Between- and within-participant variation of input signals. The majority of features related to cardiac activity as well as skin temperature vary more between participant than within the same participant. For each feature, we compare the variation in the average value per participant to the average variation per participant. Higher between-participant variation that within-participant variation makes interpretation difficult.

Fig 5 highlights between- and within-participant standard deviation for HR-, HRV- and TEMP-related features. In 18 out of 25 cases, including all HR-related features, we found a higher between-participant standard deviation than the within-participant standard deviation. When the between-participant standard deviation is higher than the within-participant standard deviation, this translates to the mean value of the feature per participant varying on average greater than the feature per individual participant. Per-participant normalization is thus required to detect more subtle changes in features such as minimum, mean and maximum heart rate collected during the awake- and the asleep-phase and model their effect on perceived sleep quality.

## 5. Results

The metrics we report in this section are based on perfectly balanced test sets. We obtained these from the datasets described above by removing the over-represented class via downsampling. For each dataset, we modeled absolute perceived sleep quality using the recorded feature values and normalized perceived sleep quality using normalized features.

### 5.1 Binary classification of absolute sleep quality

Table 2 lists the results of different modeling techniques for absolute perceived sleep quality. Using dataset B, we observed the highest balanced accuracy of 64% and the highest F1 score of 63% outperforming the work by Gashi et al. [9] by 23% and 14%, respectively. On dataset A, we observe the highest AUC of 0.70 for absolute perceived sleep quality.

**Table 2:** Results for modeling absolute perceived sleep quality.

method	model	dataset	BA	F1	AUC
Gashi et al. [9]	GB	-	0.53	0.57	-
ours	TE	A	0.63	0.59	0.70
ours	TE	B	0.64	0.63	0.67

Without prior knowledge about the participants, Gashi et al. [9] achieve the highest performance (in terms of F1 score) using a gradient boosting (GB) classifier only using features related to wrist movement. Including features about electrodermal activity and skin temperature decreases their model’s performance. They do not consider features about cardiac activity. We use a tree ensemble (TE) classifier on the two dataset we constructed (dataset A and B) with features containing information about cardiac activity, electrodermal activity, skin temperature and wrist movement. We compare model performance in terms of balanced accuracy (BA), F1 score, and area under the curve (AUC).

### 5.2 Binary classification of normalized sleep quality

After normalizing perceived sleep quality and input features, we achieved similar accuracies and AUC values. However, with a balanced accuracy of 0.70 and an AUC of 0.76, the accuracy and F1-score achieved by the best performing model was higher than what we previously observed for any model for absolute perceived sleep quality. We evaluated three different models across different subsets of all available features as listed in Table 3: a tree ensemble method (TE) and two ‘glass-box models’ in form of a GLM and a GAM.

**Table 3:** Ablation study for modeling normalized perceived sleep quality.

		CAR		CAR		CAR								CAR			
	features:	TEMP		TEMP				TEMP				TEMP					
		EDA				EDA		EDA		EDA							
model	dataset	BA	AUC	BA	AUC	BA	AUC	BA	AUC	BA	AUC	BA	AUC	BA	AUC	BA	AUC
TE	A	0.6	0.61	0.7	0.76	0.56	0.57	0.61	0.61	0.56	0.55	0.66	0.67	0.58	0.61	0.58	0.62
TE	B	0.62	0.6	0.61	0.62	0.57	0.6	0.58	0.59	0.54	0.55	0.61	0.6	0.59	0.58	0.55	0.58
GAM	A	0.6	0.61	0.58	0.62	0.58	0.6	0.59	0.61	0.61	0.64	0.62	0.61	0.58	0.61	0.62	0.64
GAM	B	0.59	0.6	0.59	0.61	0.56	0.59	0.56	0.58	0.55	0.6	0.57	0.6	0.56	0.6	0.58	0.63
GLM	A	0.63	0.69	0.59	0.6	0.61	0.59	0.62	0.62	0.61	0.63	0.62	0.6	0.59	0.61	0.62	0.65
GLM	B	0.56	0.6	0.58	0.62	0.59	0.61	0.56	0.58	0.57	0.59	0.56	0.6	0.57	0.62	0.59	0.62

On dataset A and B, we performed an ablation study comparing a generalized additive model (GAM), a generalized linear model (GLM) and a tree ensemble (TE) across different combinations of features. Features derived based on wrist movement were included in any model. In the model on the very left the three feature groups related cardiac activity (CAR), skin temperature (TEMP) and electrodermal activity (EDA) were included. On the very right, all three feature groups are removed. We compare model performance in terms of balanced accuracy (BA), and area under the curve (AUC).

#### 5.2.1 Performance based on different datasets

We observe the highest performances on dataset A. There are a few cases, however, where the TE, GAM, or GLM perform better on dataset B than dataset.

#### 5.2.2 Performance using different models

To analyze how features about cardiac activity, TEMP and EDA can improve model performance in addition to features derived based on actigraphy alone, we conducted an ablation study across all subsets of EDA, TEMP and cardiac activity features. Features based on actigraphy have been used extensively to also model objective sleep quality metrics and are included in all models.

We observed the highest overall balanced accuracy of 0.70 when including features about cardiac activity and TEMP in addition to actigraphy-based features but excluding EDA-related features. This was achieved using the tree ensemble (TE). The GAM and GLM outperformed the TE when all features regarding EDA, TEMP, and cardiac activity were removed. For the tree-ensemble, using TEMP-related features in combination with cardiac activity features or EDA-features, or using TEMP-related features alone performed better than using EDA-related features alone, cardiac activity features alone, a combination of the cardiac activity and EDA features or all features together.

### 5.3 Feature importance & partial dependence plots

**Fig 6:**
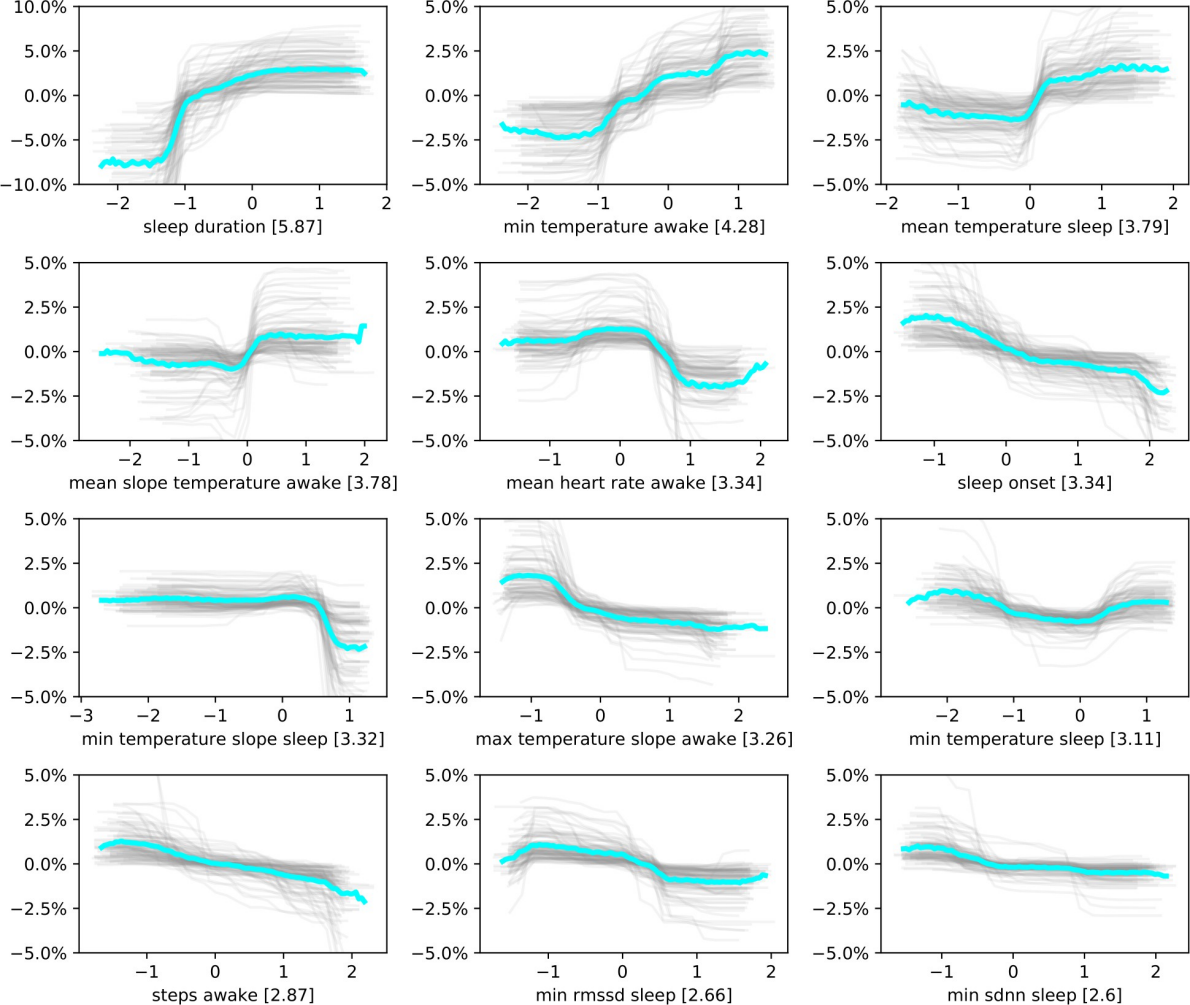
Partial dependence plots (PDPs) for the 12 most import features of the tree ensemble classifier for normalized perceived sleep quality. The plots were calculated on dataset A, using the optimal performing classifier from Table 3. We bootstrapped the partial dependence plots 100 times. Gray lines correspond to one partial dependence plot centered around 0. The bright blue line corresponds to the average across all bootstrapped samples. The x-axis reflects the variable normalized per participant. The y-axis reflects the calculated increase in the probability of observing sleep quality higher than the participants’ averages.

To analyze how single features relate to normalized perceived sleep quality, we calculated feature importances (average change in probability of high sleep quality due to change in input feature) using the best-performing model in Table 3. We thus included all features apart from EDA-related features. Furthermore, we constructed partial dependence plots (PDPs) for the 12 most important features (Fig 6). We bootstrapped the classifier 100 times to derive the stability of the partial dependence plots.

Sleep duration was the feature with the highest importance, followed by minimum skin temperature while awake and mean skin temperature while asleep. In total, 6 out of the 12 most important features are related to skin temperature. In addition to sleep onset and actigraphy counts while awake, three features related to cardiac activity were among the 12 most important features.

All of the PDPs show a clear trend. The classifier associates higher sleep duration and shorter sleep onset with a higher chance of observing high normalized perceived sleep quality. The PDPs for skin temperature indicate that the classifier calculates increased chances of observing higher normalized perceived sleep quality when average skin temperature while awake and minimal skin temperature while asleep is increased, and when minimal skin temperature while asleep is close to an individual’s average. An increase in the average change in skin temperature over 5-minute windows while awake is calculated to increase the chances of higher normalized perceived sleep quality, while an increase in the maximum change of skin temperature over any 5-minute interval is calculated to decrease the chances of observing high normalized perceived sleep quality. The classifier further associates higher minimal RMSSD and SDNN while asleep with decreased chances of observing high normalized perceived sleep quality. An average heart rate while awake that deviates from an individual’s mean is calculated to lower the chances high normalized perceived sleep quality by the tree-ensemble classifier. Furthermore, an increased number of activity counts is associated with lower chances of observing high normalized perceived sleep quality.

### 5.4 Analysis of misclassified observation

**Fig 7:**
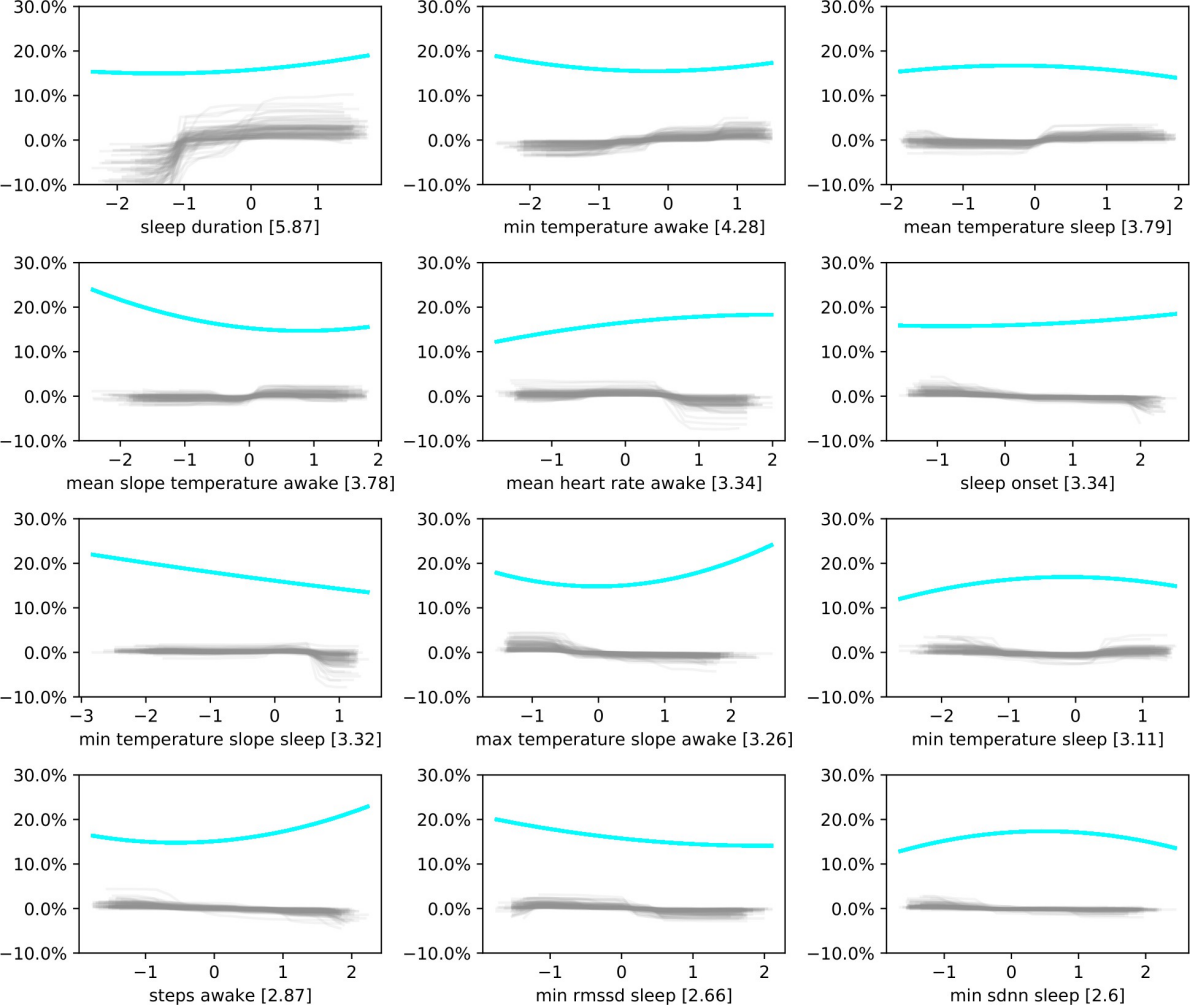
Misclassification error plots for the 12 most important features of the tree ensemble classifier for normalized perceived sleep quality. To assess errors in the calculated decision functions per feature, we calculated the error rate per feature value for the 12 most important features. Blue lines are smoothed misclassification rates. A clear trend indicates that the decision function of the classifier is not properly adjusted for new unseen observations but might be overfitting on noise. Grey lines show the bootstrapped partial dependence plots as in Fig 6.

The highest balanced accuracy we observed was 70%. On a perfectly balanced test set, 30% of observations this get predicted incorrectly. To better understand what observations the classifier predicts incorrectly, we plotted the proportion of misclassified observations per feature value in Fig 7. We focused on the 12 most important features as calculated for Fig 6. While the proportions seem stable across different values for normalized sleep duration, minimal skin temperature while awake, minimal skin temperature while asleep and sleep onset, the other plots show clearer trends. The proportion of incorrectly classified observations, for instance, increases with decreasing average change in temperature while awake, or decreasing minimal temperature change while asleep. This indicates incorrectly calculated decision functions of the classifier based on these features.

**Fig 8:**
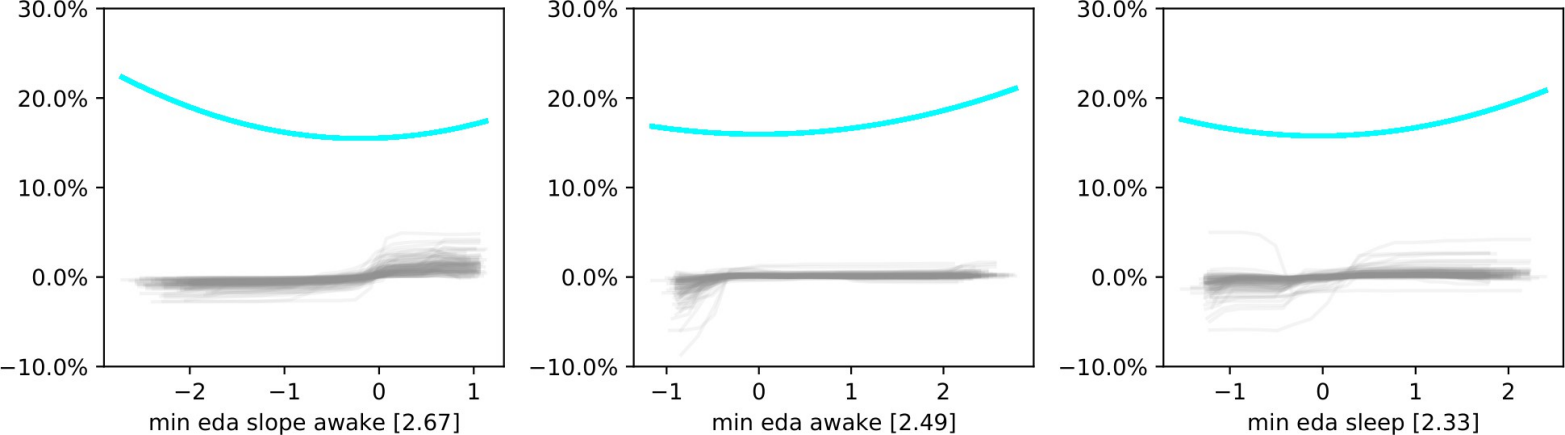
Misclassification error plots for the three most important features related to electrodermal activity (EDA). Blue lanes show the smoothed misclassification rate per feature value. Removing EDA-related features from the feature set improved model performance. We show the misclassification error plots for the three most important EDA-features when the classifier is trained on all available features including EDA-related features. The clear trends in the error plots indicate overfitting to noise and offer an explanation why their removal was beneficial for performance.

Fig 8 displays similar plots for the three most important EDA-related features when the tree-ensemble is trained on all available features. All three plots show a clear trend, indicating potential overfitting on EDA-related features and a miscalculation of the effect of change in these features on the chances of observing high normalized perceived sleep quality.

## 6. Discussion

In this paper we proposed a novel approach to model perceived sleep quality for consecutive days based on wearable sensor data. We outperform previous work using simple and interpretable features and demonstrated the need to normalize the response as well as input features. Further, our approach highlights the importance of features related to cardiac activity such as HR and HRV.

### 6.1 Comparison to prior work

In comparison to related work, our models for absolute perceived sleep quality achieved higher predictive performances independent of whether we used dataset A or B. Our tree ensemble on dataset A reduced error rates by 36%.

### 6.2 Performance on different datasets

We constructed 2 different datasets based on the M2Sleep dataset: dataset A, and dataset B. When modeling normalized perceived sleep quality, the performance of models trained on dataset A was generally higher. When modeling absolute perceived sleep quality, however, the performance was very higher on dataset B (differences in balanced accuracy of 1%). Since the dataset B contains nearly 50% more observations than dataset A, our approach utilizing the raw BVP output of the Empatica E4 has much greater real-world applicability for modeling absolute perceived sleep quality than relying on the IBIs derived by the Empatica E4. The higher performance when modeling normalized perceived sleep quality, however, shows that the features related to cardiac activity are likely to be more informative when derived based on the IBIs supplied by the Empatica E4. Few erroneously-detected heart beats can already affect computed HRV metrics [80]. Accurate detection of motion artifacts is thus of great importance and should be included into the processing stage of future work.

### 6.3 Sleep quality: As recorded vs. normalized

With modeling normalized perceived sleep quality, we have proposed a novel approach to model perceived sleep quality in intensive longitudinal studies that generates more useful predictions for users since the sleep quality threshold is chosen for each individual rather than for the whole population. This leads to more balanced per-participant distributions of the response labels as shown in Section 4.1.2. The entropy and information content for the participant is thus higher when the binary label of perceived sleep quality is chosen based on the average response of each participant. By normalizing features per participant when modeling normalized perceived sleep quality, we increased the interpretability of the results and generated more medically relevant features. Even though we achieved a higher balanced accuracy and AUC when modeling normalized perceived sleep quality and selection the best performing feature subset, the overall performance seems similar.

### 6.4 Feature-dependent performance for normalized perceived sleep quality

While assessing what performances different feature combinations achieved on dataset A modeling normalized perceived sleep quality, we observed the highest accuracies and AUC values when excluding one of the features. Removing features either related to EDA, to cardiac activity, or both groups of features, resulted in higher performance than including all available features, indicating potential overfitting to noise. We achieved the highest performance in terms of F1-score and accuracy when excluding EDA-related features. We analyzed the proportion of misclassified observations per feature and noticed a clear trend in the plots about the most important features related to EDA when all features were used to model normalized perceived sleep quality. This offers a potential explanation as to why dropping a group of features boosts performance rather than harm it. Since the proportions of misclassified observations changed across different values of the EDA features, decision functions were calculated incorrectly—likely due to overfitting on these features. The accuracy of the tree ensemble when excluding EDA-related features increased from 60% to 70%. Similarly, the AUC increased from 0.61 to 0.76. This also indicates a lower value of EDA-related features to analyze sleep, despite differences in EDA between different sleep stages [84] and its potential to collect information about periods of arousal [102]. Since we only focused on simple features, more complex EDA-related features might prove more informative when modeling perceived sleep quality.

### 6.5 Feature importance and interpretation

Through multiple restrictions, we have ensured that the results of our approach remain interpretable. We have only used simple features, a model that is explainable through partial dependence plots even though it is not a ‘glass-box model,’ and through feature normalization we have also proposed an approach that generates medically interpretable results. While the approach allows the authors of the paper to infer about potential effects of variables on perceived sleep quality, this must not hold true for medical practitioners. The following Section outlines insights generated from the PDPs based on 100 bootstrapped classifiers for normalized perceived sleep quality on the feature subset where we observed the highest performance (dataset A when excluding EDA-related features).

#### 6.5.1 Actigraphy

Using actigraphy, we derived sleep duration, sleep onset and actigraphy counts while participants were awake and asleep. Sleep duration was the most important feature used by our classifier. The bootstrapped PDPs show a clear trend indicating that short sleep duration was associated with lower perceived sleep quality. Shorter sleep onset, on the other hand, was calculated to increase the chances of high perceived sleep quality. Even though actigraphy is in common use in sleep studies, it is not exact at estimating sleep and was shown to differ on average up to 20 minutes from the ground truth [91, 92]. The calculated sleep and wake periods are thus noisy. This is consistent with existing literature and results obtained in sleep laboratories [3].

#### 6.5.2 Cardiac activity

Adding heart rate and heart rate variability to the modeling process improved the accuracy of our approach compared to existing work from Gashi et al. [9] (from 53% to 63%). This highlights the importance of information about heart rate and heart rate variability to model perceived sleep quality. However, if only features derived from actigraphy are included in the model, adding TEMP-related features results in higher model performance than adding features related to cardiac activity indicating that cardiac features are less crucial for model performance than TEMP-related features. We found average heart rate while awake to be the most import feature about cardiac activity for our classifier. The chances of high perceived sleep quality were calculated to decrease the further the average heart rate per day deviates from other day’s averages, which might be caused by increase physical activity, stress or very low physical activity throughout the day.

#### 6.5.3 Skin temperature

Features related to TEMP represented the majority of the 12 most important features. This is in accordance with the tree ensemble performing generally best if TEMP-related features were included. When only including one subset of features, the tree ensemble performed best on the subset including only TEMP-related features. An increased minimal skin temperature while awake and increased average skin temperature while asleep are calculated to increase the chances of observing high perceived sleep quality. For other TEMP-related features, we observe a clear trend when plotting the proportion of misclassified observations per feature value. The classifier might thus be in danger of overfitting on these features. This trend is especially strong for features related to the changes of skin temperature over 5-minute windows.

## 7. Limitations & future work

Our approach to model normalized perceived sleep quality is mainly limited by the required amount of labeled data in order to normalize perceived sleep quality. To normalize features and perceived sleep quality, at least two unique values have to be recorded for each feature as well as the response. Since perceived sleep quality responses are often recorded on Likert scales with a relatively small number of possible answers, for some participants this might result in a relatively high minimum number of days with data recordings. On the M2Sleep dataset [9], for instance, participant S09 reported the same perceived sleep quality response in nearly 87% of the cases. Our approach requires a minimum of two days per potential user to provide any predictions at all. A population model for absolute perceived sleep quality (for instance trained on the M2Sleep dataset), can be used from day-one for unknown individuals.

Our analysis of the drivers of perceived sleep quality on the M2Sleep dataset is limited by the limitations of the dataset and the Empatica E4 itself for our use-case. The Empatica has been validated in various studies [65–67]. However, its accuracy has been shown to be slightly below gold-standard ECG devices. Thus, heart rate and heart rate variability metrics supplied by the Empatica E4 to be treated with caution. While we disregard 5-minute windows with low signal quality, there is no absolute certainty about whether all noise introduced for instance by motion artifacts, incorrect sensor placement or possible synchronization issues is removed. When we used IBIs supplied directly by the E4, we had to remove a large proportion of data points due to no consecutive IBIs being provided across any 5-minute interval while participants were awake. This left us with only 222 observations out of a possible 463. The size of the dataset might have been increased for our use-case if participants had worn a wearable sensor for the full day rather than 4 hours before going to bed and 4 hours after waking up. This would have left more opportunities for 5-minute intervals with consecutive IBIs without motion artifacts during the awake phase and would have captured the full variability of heart rate dynamics. However, even without removing any data the M2Sleep dataset has limited generalizability to the broader population due to its small number of participants and a non-representative age distribution of 19 to 35 years. It also remains unclear if 30 days are long enough to capture the full phenomenon of perceived sleep quality. Future research will benefit from additional datasets that combine perceived sleep quality with continuously recorded biosignal (exceeding actigraphy) across multiple days or even months. Diverse, large scale longitudinal studies will be required to assess any effects calculated by our approach.

For future work, the improvement in accuracy achieved by including features related to cardiac activity highlights two points worth noting regarding the use of features about cardiac activity to model perceived sleep quality. First, as touched upon above, wearable sensors such as the Empatica E4 still struggle to record IBIs reliably enough to collect HRV data while users are awake and live their daily lives. This issue could be addressed by using different data processing techniques for the BVP signal of the Empatica E4 to manually detect the IBIs as we have shown with dataset B. However, data quality seems to have dropped due to this approach. Second, heart rate and heart rate variability data recorded while awake and asleep can greatly improve performance, which highlights their importance towards a better understanding of perceived sleep quality. In future analysis, they should thus be included.

## 8. Conclusion

In this paper we have proposed a new approach for modeling perceived sleep quality. Specifically, our addition of heart rate and heart rate variability features showed considerable improvement in predicting users’ perceived sleep quality over previous work. In our evaluation, we also showcased that a normalization of features and perceived sleep quality increases medical interpretability and the information content of predictions for participants. Due to simplistic features used in our post-hoc explainable model, our approach allows interpreting results and reveals the impact of sleep duration, previous-day activity, minimal activity of the sympathetic nervous system and minimal heart rate while asleep on normalized perceived sleep quality.

Our results highlight the benefit of modeling measures of subjective well-being, such as perceived sleep quality, using data obtained using today’s wrist-worn wearable sensors across multiple days. We believe our approach generalizes to other use-cases exceeding the modeling of perceived sleep quality. While features from users’ cardiac activity were a key enabler of our method, we also observed a drawback in their use, which was data quality and resulting usefulness. In our approach, extracting heart rate and, especially, heart rate variability led to portions of data that could not be processed—both in the metrics reported by vendor algorithms that ship on the Empatica E4 watch (241 out of 463) as well as using our processing of the device’s PPG recordings, though at much smaller dropout rates (133 of 463). Nonetheless, we believe that our method shows a promising path for the estimation of perceived sleep quality and that ongoing advancements in sensing technology and signal processing will increase the quality and availability of features in the future.

## Supporting information

S1 Graphical abstract We present a novel approach to model perceived sleep quality over consecutive nights using only simplistic and interpretable features. Our approach achieves an accuracy of 70% with an AUC of 0.76 and reduces error rates by up to 21% compared to previous work. The explainability of our model coupled with interpretable features allows us to analyze the drivers of perceived sleep quality, which revealed the impact of sleep duration, sleep onset, minimal skin temperature while awake, average skin temperature while asleep and average heart rate while awake on perceived sleep quality.
(TIF)
