# Supplementary figures and images for: Personalized interpretable prediction of perceived sleep quality: Models with meaningful cardiovascular and behavioral features
Source: PLoS One. 2024 Jul 8;19(7):e0305258. doi: 10.1371/journal.pone.0305258 (PMC11230538; doi:10.1371/journal.pone.0305258)

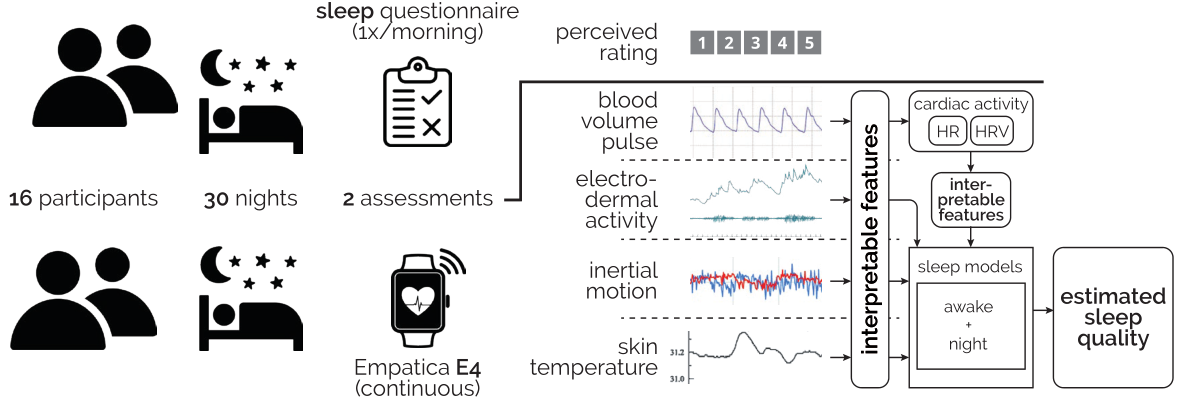

Supplement: S1 Graphical abstract — Our approach achieves an accuracy of 70% with an AUC of 0.76 and reduces error rates by up to 21% compared to previous work. The explainability of our model coupled with interpretable features allows us to analyze the drivers of perceived sleep quality, which revealed the impact of sleep duration, sleep onset, minimal skin temperature while awake, average skin temperature while asleep and average heart rate while awake on perceived sleep quality. (TIF) [file pone.0305258.s001.tif]
